# Swedish Olympic athletes report one injury insurance claim every second year: a 22-year insurance registry-based cohort study

**DOI:** 10.1007/s00167-023-07511-y

**Published:** 2023-07-15

**Authors:** Kalle Torvaldsson, Hanna Lindblom, Sofi Sonesson, Eric Hamrin Senorski, Helena Stigson, Lykke Tamm, Jörgen Sandberg, Martin Hägglund

**Affiliations:** 1 Department of Health, Medicine and Caring Sciences, Unit of Physiotherapy, Linköping University, Linköping, Sweden; 2 Sport Without Injury ProgrammE (SWIPE), Department of Health, Medicine and Caring Sciences, Linköping University, Linköping, Sweden; 3 Institute of Neuroscience and Physiology, Sahlgrenska Academy, University of Gothenburg, Gothenburg, Sweden; 4 Folksam Research, Folksam Insurance Group, Stockholm, Sweden; 5 Division of Insurance Medicine, Department of Clinical Neuroscience, Karolinska Institutet, Stockholm, Sweden; 6 Swedish Olympic Committee, Sofiatornet, Olympiastadion, Stockholm, Sweden

**Keywords:** Sports injury, Epidemiology, Olympics, Surveillance, Insurance

## Abstract

**Purpose:**

To describe injury incidence, time trends in injury incidence, and injury characteristics among Swedish Olympic athletes over 22 years based on insurance data, as a first step to inform injury preventive measures among Olympic athletes.

**Methods:**

The cohort comprised 762 elite athletes (54% males; age 26.5 ± 5.9 years) in 38 sports in the Swedish Olympic Committee support program ‘Top and Talent’ between 1999 and 2020, with total 3427 athlete-years included. Acute and gradual onset injuries were reported to the insurance registry by the athletes’ medical staff.

**Results:**

A total of 1635 injuries in 468 athletes were registered. The overall injury incidence was 47.7 injuries/100 athlete-years (one injury per athlete every second year). An increasing trend in injury incidence was observed in the first decade 2001 to 2010 (annual change 6.0%, 95% CI 3.3–8.8%), while in the second decade 2011 to 2020 no change was evident (0.4%, 95% CI − 1.9 to 2.7%). Gymnastics, tennis, and athletics had the highest incidence (100.0, 99.3, and 93.4 injuries/100 athlete-years, respectively). Among sport categories, mixed and power sports had the highest incidence (72.8 and 69.5 injuries/100 athlete-years, respectively). Higher incidences were seen in the younger age groups (≤ 25 years) in mixed and skill sports. The injury incidence was comparable between male and female athletes, and summer and winter sports. Most injuries occurred in the lower limb, and specifically the knee (24%), foot/ankle (15%) and spine/pelvis (13%).

**Conclusion:**

The results on injury patterns in different sports and age groups may guide preventive focus for health and performance teams working with Olympic athletes.

**Level of evidence:**

II.

**Supplementary Information:**

The online version contains supplementary material available at 10.1007/s00167-023-07511-y.

## Introduction

Injuries entail major challenges for sports teams and athletes, as they have a negative impact on athletes’ health, sports performance, and success [5, 6, 12, 26]. Structured long-term injury surveillance is fundamental to better understand the injury burden and pattern, to generate ideas for injury prevention, and to evaluate the effects of interventions [24]. Long-term surveillance programmes have been established among elite athletes in sports such as football, rugby, and athletics and have provided increased knowledge of the injury burden in these sports [7, 8, 11, 14, 27]. For instance, based on FIFA injury surveillance there is evidence of reduced incidence of head injuries in professional football after introduction of a rule change penalizing direct and deliberate elbows to head with a red card [2].

For the 2008 Olympic Games, the International Olympic Committee implemented a health surveillance programme which has been conducted ever since [9, 10, 15, 19–22]. During the summer Olympic Games of 2016 and 2020 and the winter Olympic Games of 2014 and 2018, 8–12% of athletes suffered an injury [19–22]. This surveillance programme, however, only captures injuries occurring during the Olympic Games, while the injury rate and pattern outside this period is largely unknown. Injuries occurring outside the Olympic Games period could have an impact on athletes’ preparations for the Olympic Games, and more knowledge about injuries may help support athletes in their preparations, potentially reducing the injury burden and allowing better performance. Additionally, surveillance programmes may act as a first step to inform injury preventive measures. Many National Olympic Committees have support programmes for their Olympic candidates. In Norway, for example, a health monitoring programme was implemented in 2011 for the national Olympic and Paralympic candidates, which has provided further insights about injuries outside the Olympic Games period [4].

Insurance registries have been used to collect injury surveillance data [13, 16, 29, 30]. Most athletes in Sweden are insured by the same insurance company, where acute sport injuries have been registered consistently for over 20 years [30]. Previously this registry has been used to study sports injuries at a national level [29], but it has not been used to study injuries specifically occurring among elite Olympic athletes. The aims of this study were to: (1) describe the injury incidence and analyse time trends in injury incidence; and (2) describe injury types and locations, in different age groups, sexes, and sports among Swedish Olympic athletes based on insurance data.

## Materials and methods

### Study design

This was a cohort study based on insurance registry data from the Swedish insurance company Folksam Insurance Group (Folksam). All data were handled confidentially in line with the Declaration of Helsinki. The study was approved by the Swedish Ethical Review Authority (reg. no 2022-00438-01). The study was reported according to the Strengthening the Reporting of Observational Studies in Epidemiology and the Sport Injury and Illness Surveillance extension [1, 25].

### Population

The study population includes Swedish elite male and female athletes, covering a wide range of sports, within the Swedish Olympic Committee (SOC) support programme Top and Talent (Table 1). Inclusion criteria were all athletes that had participated in Top and Talent between January 1999 and December 2020. No exclusion criteria were applied. Top and Talent was initiated in 1998 and is an individually customised support programme for athletes with potential medal capacity in the Olympic Games, with a plan for the athlete to reach top international positions within 3–6 years of entering the programme. Athletes are accepted to the programme based on either top performance in international competitions or a talent selection [23]. Approximately 30% of the athletes in Top and Talent reach medal capacity in the Olympic Games or world championship after 4–6 years of entering the programme. Athletes in Top and Talent are provided with, among other things, medical, coaching, training, and nutritional support, and all athletes have health insurance from Folksam [23]. A total of 762 athletes (412 males, 350 females) in 38 sports participated in Top and Talent between 1999 and 2020 (Table 2). Mean ± SD age of the included athletes was 26.5 ± 5.9 years. On average, 172 ± 23 athletes (range 121–217) participated in the programme each year during the observation period. Duration of participation was median 4 years (IQR 2–6), with total of 3427 athlete-years included.

**Table 1:** Data collection and definitions

Data collection	
For each insured athlete	Age, sex, sport, sport category (skill/power/mixed/endurance), competitive season (summer/winter sport), number of years participating in Top and Talent
For each injury	Injury type, body region, location, date of injury, athlete´s age at time of injury
Sport category definition [17] and included sports	
Skill	*Definition*: Achievement depends mainly on technical or bodily skill
	*Included sports*: Archery, curling, diving, equestrian, golf, sailing, shooting, skateboarding, ski jumping, table tennis
Power	*Definition*: Achievement depends mainly on explosive muscle power
	*Included sports*: Alpine skiing, figure skating, freestyle skiing, gymnastics, judo, karate, snowboard, sport climbing, taekwondo, weightlifting, wrestling
Mixed	*Definition*: Alternating phases of dynamic and/or static work and recovery, with variations in duration and intensity depending on sport and athlete’s role
	*Included sports*: Athletics, badminton, basketball, boxing, fencing, handball, modern pentathlon, tennis, volleyball
Endurance	*Definition*: Prolonged and intensive dynamic and/or static work
	*Included sports*: Biathlon, canoe, cross-country skiing, cycling, rowing, speed skating, swimming, triathlon
Injury definition	Any physical damage with a sudden or gradual onset that met the insurance eligibility criteria and that occurred while the athlete participated in Top and Talent

**Table 2:** Number of athletes in top and talent by sport category, sport, and sex

Sport category/sport	Total population		Male		Female	
	n	Athlete-years^a^	n	Athlete-years^a^	n	Athlete-years^a^
Skill sports	200	921.5	117	565	83	356.5
Archery	10	42.5	5	24	5	18.5
Curling	41	192	17	89	24	103
Diving	4	23	2	5.5	2	17.5
Equestrian	39	193	17	91.5	22	101.5
Golf	10	42	5	21	5	21
Sailing	44	187	30	134.5	14	52.5
Shooting	28	130.5	21	96	7	34.5
Skateboarding	2	5	2	5	0	0
Ski jumping	2	7	2	7	0	0
Table tennis	20	99.5	16	91.5	4	8
Power sports	195	948.5	108	524	87	424.5
Alpine skiing	37	233.5	15	106.5	22	127
Figure skating	11	58	4	26	7	32
Freestyle skiing	41	162	30	116.5	11	45.5
Gymnastics	7	29	2	8	5	21
Judo	20	104	11	64	9	40
Karate	1	2	0	0	1	2
Snowboard	26	115.5	19	82	7	33.5
Sport climbing	1	0.5	1	0.5	0	0
Taekwondo	14	45	5	13.5	9	31.5
Weightlifting	3	4	2	2.5	1	1.5
Wrestling	34	195	19	104.5	15	90.5
Mixed sports	173	627.5	92	347	81	280.5
Athletics	63	290	33	152	30	138
Badminton	19	61	11	36	8	25
Basketball	8	25.5	0	0	8	25.5
Boxing	21	64.5	15	46	6	18.5
Fencing	12	59	7	30	5	29
Handball	11	24.5	7	18.5	4	6
Modern pentathlon	5	20.5	3	16	2	4.5
Tennis	21	68.5	12	43.5	9	25
Volleyball	13	14	4	5	9	9
Endurance sports	194	929.5	95	495.5	99	434
Biathlon	22	89	11	54	11	35
Canoe	21	131	15	93.5	6	37.5
Cross-country skiing	55	283.5	28	167.5	27	116
Cycling	21	99	9	42.5	12	56.5
Rowing	10	40	3	15	7	25
Speed skating	7	33	7	33	0	0
Swimming	55	234	20	80	35	154
Triathlon	3	20	2	10	1	10
Total	762	3427	412	1931.5	350	1495.5

^a^Athlete-years are defined as the number of years an athlete participated in Top and Talent. If an athlete only participated half a year (spring or autumn) they were given a value of 0.5

### Data collection

Injury insurance claims were reported to the registry prospectively between 1999 and 2020 by a physician or physiotherapist from SOC via the athletes’ health insurance, after a median of 5 days (IQR 0–22) from date of injury. Only injuries occurring while the athletes participated in Top and Talent were registered. An administrator from Folksam was responsible for recording the injury in the registry. Injuries were diagnosed according to an insurance-specific coding system. The registry contained data about the insured athletes and about each injury. Details on the collected data are presented in Table 1. Injury data were extracted from the insurance registry in September 2022. Initial data processing and database construction was done by Folksam, and the data received for research and analysis were pseudonymised. The database was checked for errors by the first author, and any mismatching information (e.g. mismatched injury location and injury type) were checked against the original insurance claim record by Folksam, and corrected where possible.

### Definition of injury

The health insurance covers both injuries and illnesses, but only injuries were included in this study. An eligible injury, as per the insurance policy, was defined as any physical damage with a sudden or gradual onset occurring while the athlete participated in Top and Talent. Pre-existing injuries present prior to start of the insurance were not included. Multiple injuries sustained by an athlete in a single event were recorded as one injury with multiple injury types or locations. Injury type was classified into eight different categories. Due to irregular reporting in the insurance registry, injuries reported as joint/ligament sprains, dislocations, and muscle/tendon strains and ruptures were combined by the authors to the same injury type category for descriptive statistics, named ‘soft tissue/joint injury’. The other injury type categories were contusion, fracture/bone injury, inflammation, concussion, laceration/abrasion, multiple injury types, and ‘unspecified’. Injuries were classified to a body region and location based on the reports made to the insurance registry.

### Statistical analysis

Data were summarised using descriptive statistics, with mean, median, frequencies, standard deviations (SD), interquartile ranges (IQR) and/or 95% confidence intervals (CI), as appropriate. Incidence and characteristics (i.e. type and location) of injuries were presented for the total population and stratified for subgroups based on sex (male, female), competitive season (summer and winter sports), sport category (skill, power, mixed, endurance) [17], and age group (≤ 20, 21–25, 26–30, 31–35, 36–40, ≥ 41 years). Injury incidence and characteristics were presented only for individual sports with more than five athletes due to confidentiality reasons. Injury incidence was calculated as an incidence rate: number of injuries divided by number of athlete-years multiplied by 100 and expressed as number of injuries per 100 athlete-years. ‘Athlete-years’ was defined as the number of years an athlete participated in Top and Talent. If an athlete only participated half a year (spring or autumn) they were given a value of 0.5 years. All injuries (new and recurrent) and athlete-years were aggregated per athlete within each subgroup. This means that for age group specific injury numbers the same athlete can be included in multiple age groups depending on the athletes’ age at the exposure (athlete-year) and time of injury. Time trend analyses were performed for the 2001 to 2020 period with Poisson regression using generalized linear models with number of injuries as the dependent variable, year as covariate, and with natural log of the exposure variable (athlete-years) as offset variable. The model-based estimator was used for the covariance matrix and Wald statistics was used to calculate the p-value and 95% CI. The first 2 years (1999–2000) were excluded in the time trend analysis due to suspected low awareness about the health insurance when it first was released. Time trend analyses were performed for the full 20-years period for the total population and split by sex, as decided a priori. In addition, statistical time trend analyses were conducted post hoc for the first and second 10-years periods separately since ocular analysis indicated varying trends in the two decades. Time trends in injury incidence are expressed as the annual percent change between years with a 95% CI. The significance level was set at p < 0.05. All statistical analyses were performed using IBM SPSS Statistics (version 29.0, Armonk, New York, USA).

## Results

### Injury incidence

In total, 1635 injuries sustained by 468 athletes (61.4% of all athletes) were registered (median 2 injuries per athlete (IQR 1–5; range 1–21)). The overall incidence was 47.7 injuries/100 athlete-years, with 46.4 and 49.3 injuries/100 athlete-years among male and female athletes, respectively. The highest incidence per individual sport was seen in gymnastics, tennis, and athletics (100.0, 99.3, and 93.4 injuries/100 athlete-years, respectively). Injury incidence in the total population and for subgroups is presented in Tables 3 and 4, and Online Resource 1, and incidence by injury type and location is presented in Table 5.

**Table 3:** Injury incidence and number of injuries in the total population and subgroups

	Incidence (95% CI)			Injuries (n)		
	Total	Male	Female	Total	Male	Female
Total population	47.7 (45.5–50.1)	46.4 (43.5–49.6)	49.3 (45.9–53.0)	1635	897	738
Competitive season						
Summer sports	49.3 (46.4–52.2)	47.2 (43.5–51.2)	51.8 (47.6–56.5)	1110	590	520
Winter sports	44.7 (41.1–48.7)	45.0 (40.3–50.4)	44.3 (38.8–50.6)	525	307	218
Sport category						
Skill	25.8 (22.7–29.3)	27.3 (23.3–31.9)	23.6 (19.0–29.2)	238	154	84
Power	69.5 (64.4–75.0)	69.3 (62.5–76.8)	69.7 (62.2–78.1)	659	363	296
Mixed	72.8 (66.4–79.8)	69.2 (60.9–78.5)	77.4 (67.7–88.4)	457	240	217
Endurance	30.2 (26.9–34.0)	28.3 (23.9–33.3)	32.5 (27.5–38.3)	281	140	141
Age group^a^						
≤ 20 years	61.5 (54.0–70.0)	65.8 (54.2–79.9)	58.4 (49.1–69.5)	229	102	127
21–25 years	53.5 (49.7–57.6)	50.6 (45.8–56.0)	57.1 (51.3–63.5)	717	377	340
26–30 years	47.0 (43.1–51.4)	46.4 (41.3–52.0)	48.0 (41.9–55.0)	493	286	207
31–35 years	34.3 (28.8–40.8)	38.9 (31.8–47.7)	25.9 (18.5–36.2)	127	93	34
36–40 years	30.6 (22.9–40.8)	26.0 (16.8–40.3)	35.4 (24.1–52.0)	46	20	26
≥ 41 years	19.7 (13.1–29.7)	24.5 (15.6–38.4)	10.3 (3.8–27.3)	23	19	4

Injury incidence is reported per 100 athlete-years

*CI* confidence interval

^a^Data on age is missing for 14 athletes

**Table 4:** Injuries, injury incidence and most injured locations by sport

Sport^a^	Competitive season	Sport category	Injuries (n)	Incidence (95% CI)	Most injured locations
Gymnastics	Summer	Power	29	100.0 (69.5–143.9)	Knee (27.6%); Foot/ankle (24.1%); Spine/pelvis (13.8%)
Tennis	Summer	Mixed	68	99.3 (78.3–125.9)	Knee (19.1%); Hand/wrist (14.7%); Foot/ankle (13.2%)
Athletics	Summer	Mixed	271	93.4 (83.0–105.3)	Foot/ankle (24.4%); Thigh/hip/groin (22.1%); Knee (15.5%)
Judo	Summer	Power	96	92.3 (75.6–112.7)	Knee (29.2%); Shoulder (22.9%); Hand/wrist (11.5%)
Wrestling	Summer	Power	172	88.2 (76.0–102.4)	Knee (34.3%); Shoulder (11.0%); Spine/pelvis (10.5%)
Taekwondo	Summer	Power	38	84.4 (61.4–116.1)	Foot/ankle (36.8%); Hand/wrist (18.4%); Knee (13.2%)
Freestyle skiing	Winter	Power	117	72.2 (60.3–86.6)	Knee (38.5%); Spine/pelvis (16.2%); Shoulder (9.4%)
Boxing	Summer	Mixed	40	62.0 (45.5–84.5)	Hand/wrist (40.0%); Shoulder (12.5%); Knee (10.0%)
Figure skating	Winter	Power	30	51.7 (36.2–74.0)	Foot/ankle (56.7%); Thigh/hip/groin (20.0%); Knee (10.0%)
Alpine skiing	Winter	Power	118	50.5 (42.2–60.5)	Knee (44.9%); Spine/pelvis (16.9%); Hand/wrist (9.3%)
Fencing	Summer	Mixed	29	49.2 (34.2–70.7)	Lower leg (20.7%); Foot/ankle, Knee, Thigh/hip/groin (all 13.8%)
Snowboard	Winter	Power	52	45.0 (34.3–59.1)	Knee (26.9%); Foot/ankle, Shoulder (both 17.3%)
Volleyball	Summer	Mixed	6	42.9 (19.3–95.4)	Knee (50.0%); Abdomen, Foot/ankle, Spine/pelvis (all 16.7%)
Table tennis	Summer	Skill	42	42.2 (31.2–57.1)	Foot/ankle, Shoulder (both 23.8%); Spine/pelvis (14.3%)
Canoe	Summer	Endurance	55	42.0 (32.2–54.7)	Shoulder (21.8%); Spine/pelvis (20.0%); Elbow (16.4%)
Badminton	Summer	Mixed	25	41.0 (27.7–60.7)	Foot/ankle (28.0%); Knee (20.0%); Spine/pelvis (16.0%)
Cross-country skiing	Winter	Endurance	111	39.2 (32.5–47.2)	Knee (20.7%); Spine/pelvis (18.9%); Foot/ankle (15.3%)
Curling	Winter	Skill	63	32.8 (25.6–42.0)	Knee (23.8%); Spine/pelvis (20.6%); Shoulder (15.9%)
Sailing	Summer	Skill	53	28.3 (21.7–37.1)	Spine/pelvis (20.8%); Hand/wrist, Knee (both 15.1%)
Biathlon	Winter	Endurance	25	28.1 (19.0–41.6)	Knee (24.0%); Lower leg, Shoulder, Thigh/hip/groin (all 12.0%)
Basketball	Summer	Mixed	7	27.5 (13.1–57.6)	Foot/ankle, Lower leg (both 28.6%); Knee, Spine/pelvis, Thigh/hip/groin (all 14.3%)
Cycling	Summer	Endurance	27	27.3 (18.7–39.8)	Knee (33.3%); Shoulder (14.8%); Foot/ankle, Hand/wrist, Spine/pelvis (all 11.1%)
Speed skating	Winter	Endurance	8	24.2 (12.1–48.5)	Knee (50.0%); Hand/wrist, Lower leg, Shoulder, Spine/pelvis (all 12.5%)
Rowing	Summer	Endurance	7	17.5 (8.3–36.7)	Knee, Spine/pelvis (28.6%); Chest/thorax, Shoulder, Thigh/hip/groin (all 14.3%)
Handball	Summer	Mixed	4	16.3 (6.1–43.5)	Knee (75.0%); Shoulder (25.0%)
Shooting	Summer	Skill	20	15.3 (9.9–23.8)	Shoulder (40.0%); Knee (25.0%); Hand/wrist, Lower leg (both 10.0%)
Equestrian	Summer	Skill	23	11.9 (7.9–17.9)	Shoulder (21.7%); Knee, Spine/pelvis, Thigh/hip/groin (all 17.4%)
Golf	Summer	Skill	5	11.9 (5.0–28.6)	Abdomen, Foot/ankle, Hand/wrist, Knee, Multiple locations (all 20.0%)
Archery	Summer	Skill	5	11.8 (4.9–28.3)	Shoulder (40.0%); Spine/pelvis, Thigh/hip/groin, Upper limb (unspecified) (all 20.0%)
Swimming	Summer	Endurance	27	11.5 (7.9–16.8)	Shoulder (33.3%); Spine/pelvis (22.2%); Knee (18.5%)
All sports			1635	47.7 (45.5–50.1)	Knee (24.2%); Foot/ankle (15.2%); Spine/pelvis (13.1%)

Injury incidence is reported per 100 athlete-years

*CI* confidence interval

^a^Sports with ≤ 5 athletes are not presented due to confidentiality reasons (diving, karate, modern pentathlon, skateboarding, ski jumping, sport climbing, triathlon, weightlifting)

**Table 5:** Injury incidence and number of injuries by injury type and location

	Incidence (95% CI)			Injuries (n)		
	Total	Male	Female	Total	Male	Female
Injury type						
Soft tissue/joint injury	11.2 (10.1–12.4)	10.5 (9.2–12.1)	12.1 (10.5–14.0)	384	203	181
Contusion	2.3 (1.8–2.8)	2.5 (1.9–3.3)	2.0 (1.4–2.9)	78	48	30
Fracture/bone injury	2.0 (1.5–2.5)	2.2 (1.6–2.9)	1.7 (1.1–2.5)	67	42	25
Inflammation	1.5 (1.2–2.0)	1.6 (1.1–2.2)	1.5 (1.0–2.2)	52	30	22
Concussion	0.3 (0.1–0.5)	0.1 (0.0–0.4)	0.5 (0.2–1.0)	9	2	7
Laceration/abrasion	0.6 (0.4–1.0)	0.7 (0.4–1.2)	0.5 (0.3–1.1)	22	14	8
Multiple injury types	0.2 (0.1–0.4)	0.3 (0.1–0.6)	0.1 (0.0–0.5)	7	5	2
Unspecified	29.6 (27.9–31.5)	28.6 (26.3–31.1)	31.0 (28.3–33.9)	1,016	553	463
Injury location						
Head and neck	1.5 (1.1–1.9)	1.1 (0.7–1.7)	1.9 (1.3–2.7)	50	22	28
Head	0.7 (0.4–1.0)	0.6 (0.4–1.1)	0.7 (0.4–1.3)	23	12	11
Neck	0.8 (0.5–1.1)	0.5 (0.3–1.0)	1.1 (0.7–1.8)	27	10	17
Upper limb	11.2 (10.1–12.4)	11.6 (10.2–13.2)	10.7 (9.2–12.5)	384	224	160
Shoulder	5.1 (4.4–5.9)	5.5 (4.5–6.6)	4.5 (3.6–5.8)	174	106	68
Upper arm	0.1 (0.0–0.3)	0.1 (0.0–0.4)	0.1 (0.0–0.5)	4	2	2
Elbow	1.5 (1.2–2.0)	1.7 (1.2–2.3)	1.4 (0.9–2.2)	53	32	21
Forearm	0.4 (0.2–0.6)	0.4 (0.2–0.8)	0.3 (0.1–0.8)	12	7	5
Hand/wrist	4.1 (3.4–4.8)	3.9 (3.1–4.9)	4.3 (3.3–5.5)	139	75	64
Upper limb (unspecified)	0.1 (0.0–0.2)	0.1 (0.0–0.4)	–	2	2	0
Trunk	7.5 (6.6–8.4)	7.9 (6.8–9.3)	6.9 (5.7–8.4)	256	153	103
Chest/thorax	1.0 (0.7–1.4)	1.0 (0.6–1.5)	1.1 (0.7–1.7)	35	19	16
Spine/pelvis	6.3 (5.5–7.2)	6.8 (5.7–8.0)	5.6 (4.5–7.0)	215	131	84
Abdomen	0.2 (0.1–0.4)	0.2 (0.1–0.5)	0.2 (0.1–0.6)	6	3	3
Lower limb	26.9 (25.2–28.7)	25.0 (22.8–27.3)	29.4 (26.7–32.2)	921	482	439
Thigh/hip/groin	4.5 (3.9–5.3)	4.2 (3.4–5.3)	4.9 (3.9–6.1)	155	82	73
Knee	11.6 (10.5–12.8)	11.4 (10.0–13.0)	11.8 (10.2–13.6)	396	220	176
Lower leg	3.3 (2.7–3.9)	2.8 (2.1–3.7)	3.9 (3.0–5.0)	112	54	58
Foot/ankle	7.3 (6.4–8.2)	6.2 (5.2–7.4)	8.6 (7.3–10.3)	249	120	129
Lower limb (unspecified)	0.3 (0.1–0.5)	0.3 (0.1–0.7)	0.2 (0.1–0.6)	9	6	3
Multiple locations	0.5 (0.3–0.8)	0.5 (0.3–1.0)	0.5 (0.2–1.0)	17	10	7
Unspecified	0.2 (0.1–0.4)	0.3 (0.1–0.7)	0.1 (0.0–0.5)	7	6	1

Injury incidence is reported per 100 athlete-years

*CI* confidence interval

### Time trends in injury incidence

**Fig. 1:**
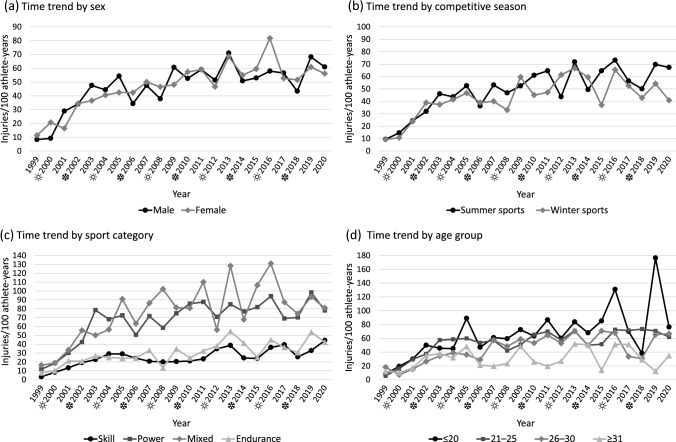
Time trend in injury incidence by sex (**a**), competitive season (**b**), sport category (**c**) and age group (**d**). Symbol next to the year illustrates whether the summer (sun symbol) or winter (snowflake symbol) Olympic Games was held in the current year. Due to small sample size in the age groups 31–35, 36–40 and ≥ 41 years, these age groups were combined to one group ≥ 31 years for visual presentation in graph. Data on age is missing for 14 athletes

The time trend analysis showed that the overall injury incidence increased by 3.0% annually over the full 20-years period between 2001 and 2020 (95% CI 2.1–3.9%, p < 0.001). Males had an 2.5% annual increased incidence (95% CI 1.4–3.7%, p < 0.001) and females an 3.7% increase (95% CI 2.3–5.1%, p < 0.001). Noteworthy was that the injury incidence increase was evident only in the first 10-years period of 2001–2010 (annual change 6.0%, 95% CI 3.3–8.8%, p < 0.001), while the second 10-year period of 2011–2020 showed no such trend (annual change 0.4%, 95% CI − 1.9 to 2.7%, (n.s.)) Year-by-year injury incidences for subgroups (sex, competitive season, sport category, and age group) are presented in Fig. 1.

### Injury characteristics

The lower limb region, and specifically the knee, had the highest injury frequency, and soft tissue/joint injury was the most common injury type category among the specified injury types. Injury characteristics are presented in Tables 4 and 5, and Online Resource 2 and 3.

## Discussion

The principal findings of the study were that the overall injury incidence among Swedish Olympic athletes increased by six percent annually between 2001 and 2010, after which the curve flattened with no change between 2011 and 2020. Athletes report approximately one injury to the insurance registry every second year, where most injuries affect the knee, foot/ankle, and spine/pelvis. There is a paucity of research on the year-round injury incidence and characteristics among Olympic athletes and the present study is, to the best of our knowledge, the first to describe injuries over a two-decade long period using insurance data.

### Injury incidence and characteristics in the total population

The injury incidence was 47.7 injuries/100 athlete-years. Compared to previous studies in Olympic athletes, this incidence rate is low. During the Olympic Games of 2014 to 2020, the injury incidence was between 5.4 and 7.8 injuries/1000 athlete-days (equal to 197.1–284.7 injuries/100 athlete-years) [19–22], and among UK Olympic summer sport athletes, an injury incidence of 1.32 injuries/athlete-year (equal to 132.0 injuries/100 athlete-years) has been reported [18]. This discrepancy may be explained by differences in data collection methods (e.g. prospective study with athlete self-report versus medical record or insurance-based registry) and injury definitions (e.g. all injuries irrespective of time-loss or medical attention versus medical attention or insurance claims), and the fact that the insurance registry used in the present study likely captures primarily more severe injuries (i.e. ‘tip of the iceberg’). The true injury incidence in this study population is probably higher, and a direct comparison to previous studies is difficult. Overall, the lower limb was the most injured body region, and the knee, followed by foot/ankle, and spine/pelvis the most injured locations, which is in line with previous studies in Olympic athletes [18–20].

### Injury incidence and characteristics in subgroups

Injury incidence was highest among power and mixed sports, possibly due to the nature and demands of these sports, which often involve high forces and athlete-to-athlete contacts. It should be noted, however, that there was a variation in injury incidence among single sports within the four sport categories. For instance, in mixed sports, the injury incidence ranged between 16.3 and 99.3 injuries/100 athlete-years, and among power sports between 45.0 and 100.0 injuries/100 athlete-years. All sports that had a higher injury incidence than the total population average belonged to the mixed or power sport categories. Previously, Ranson et al. reported that the injury incidence was highest in combat sports [18], and combat sports were included in the mixed or power sport category in the present study. Injuries to the knee and foot/ankle were prevalent among athletes in the power and mixed sport categories, indicating that these locations should be specifically targeted with injury prevention measures. In many skill and endurance sports, in addition to the knee, injuries were also frequent in the spine/pelvis and shoulder, indicating a need for other preventive measures in these types of sports.

In the present study the overall injury incidence was comparable between males and females, which is in line with previous reports during the Olympic Games [19–22]. In team sports, however, the overall injury incidence is higher among males [28], and this difference may be due to the fact that the present study mainly included individual sports. While certain knee injuries, such as anterior cruciate ligament injuries, are more common in female than in male team sport athletes [28], overall knee injuries were equally common in males and females in the present study (detailed diagnoses were not available for all knee injuries).

Comparing age groups, the highest overall injury incidence was seen among athletes ≤ 20 years, with a trend that the injury incidence decreases with higher age. This age–injury relationship was evident in the mixed and skill sport categories, but not in power and endurance sports. It should be noted, however, that comparison within single sports were not possible due to small samples, and large variations may exist. Further, there were differences in the number of athlete-years in different sports within the sport categories and age groups (Online Resource 4), and the reason for the observed age-injury relationship is unclear.

The observed variation in injury incidence and characteristics between sports and subgroups emphasises the importance of considering the contextual factors—such as environmental factors and sport and athlete characteristics—when implementing injury prevention interventions [3].

### Time trends

A significant time trend was noted with a three percent annual increase in injury incidence over the full 20-years period, which was evident in both males and females. However, when analysing time trends for the first and second 10-years periods separately, the increasing trend was evident only during the first decade of 2001 to 2010, after which the injury incidence remained fairly stable between 2011 and 2020. This indicates that there is currently no trend of increasing injury incidence in the Swedish Top and Talent athlete population. Looking at the time trends year-by-year, some variations could be seen within subgroups. For example, in winter sports, there was a tendency towards a high injury incidence the year prior to each Winter Olympic Games, and low incidence the year of the Olympic Games. While not as obvious, this tendency was also seen in summer sports in relation to the Summer Olympic Games. Based on the data it is not possible to explain the observed increasing time trend or year-by-year fluctuations in injury incidence with any certainty. It may be the result of sporting factors; e.g. changes in sporting/physical demands, competition and training schedules, or rules or equipment, but also due to methodological factors such as varying population characteristics or reporting patterns.

### Strengths and limitations

Strengths of the study include the long time period of data collection, and a large sample of athletes who had participated in Top and Talent between 1999 and 2020. In contrast to most previous research of injuries in Olympic athletes [19–22], but similar to Ranson et al. [18] and Clarsen et al. [4], the present study included also injuries sustained outside the Olympic game period, taking into consideration seasonal differences in injury rates and better reflecting the full athlete year cycle. Furthermore, the same inclusion criteria to Top and Talent were used over the 22 years, and the injury definition remained consistent over the years, allowing for comparable data over time. Another strength is the inclusion of sport-specific injury data, also providing data on injuries in sports with very little, or non-existent, previous research. To allow for meaningful comparison between groups with sufficient samples, sports and athletes were classified into subgroups. A limitation of this approach is that sports and athletes within the same subgroup may differ, and certain sports or athletes may be responsible for the majority of injury cases within the group, thus skewing the data. For example, within the mixed sport category in the present study, athletics was responsible for approximately 59% of injuries. Also, some sports, such as athletics, consist of different disciplines with varying injury burdens and patterns.

Another limitation is that many injuries were registered in the insurance registry as unspecified for injury type. Information about these injuries is therefore limited, and it is possible that the proportion of injuries in specific injury types would be different if all injuries had more specified information. More specific injury registration is important to increase the quality of the registry and to allow more definitive conclusions about injury rates and patterns.

Registry data from 1999, shortly after the inception of Top and Talent in 1998, was included. Due to low awareness of the health insurance in the initial years and likely some missing injury data, this results in underestimation of the injury incidence in these years, and to a smaller extent also the overall injury incidence. It is likely that insurance registry data includes primarily more severe injuries. Minor injuries—for example certain gradual onset injuries, or injuries not resulting in time-loss or affecting athlete performance—may go unreported to the insurance company. Given the insurance registry-based data collection, the generalisability of the findings to other populations and surveillance methods is unclear. Gradual onset injuries and non-time loss injuries dominate the injury pattern in Olympic sports [4, 19, 20], and gathering information about these injuries in a structured and systematic manner is important to get a complete understanding of injuries in Olympic athletes.

## Conclusions

The overall injury incidence increased by six percent annually between 2001 and 2010, after which the curve flattened with no change between 2011 and 2020. Swedish Olympic athletes report approximately one injury to the insurance registry every second year, where most injuries affect the knee, foot/ankle, and spine/pelvis. The highest injury incidence was seen in gymnastics, tennis, and athletics, and for specific subgroups, in mixed and power sports and younger age groups. Injury prevention interventions should be a high priority in these sports and subgroups. The findings may support health and performance teams working with Olympic athletes in their work to improve athlete health, by highlighting incidence rates and common injury locations in different subgroups and sports.

## Supplementary Information

Below is the link to the electronic supplementary material.

Supplementary file1 (PDF 169 KB)

Supplementary file2 (PDF 215 KB)

Supplementary file3 (PDF 155 KB)

Supplementary file4 (PDF 109 KB)

Supplementary file5 (PDF 90 KB)

Supplementary file6 (PDF 565 KB)

## Data Availability

The data that support the findings of this study are available from the corresponding author upon reasonable request.

## Abbreviations

CI: Confidence interval
Folksam: Folksam Insurance Group
IQR: Interquartile range
SD: Standard deviation
SOC: Swedish Olympic Committee
