# Supplementary material for: Swedish Olympic athletes report one injury insurance claim every second year: a 22-year insurance registry-based cohort study
Source: Knee Surg Sports Traumatol Arthrosc. 2023 Jul 15;31(10):4607–17. doi: 10.1007/s00167-023-07511-y (PMC10471666; doi:10.1007/s00167-023-07511-y)
Supplement: Supplementary file 1 — Supplementary file1 (PDF 169 KB) [file 167_2023_7511_MOESM1_ESM.pdf]

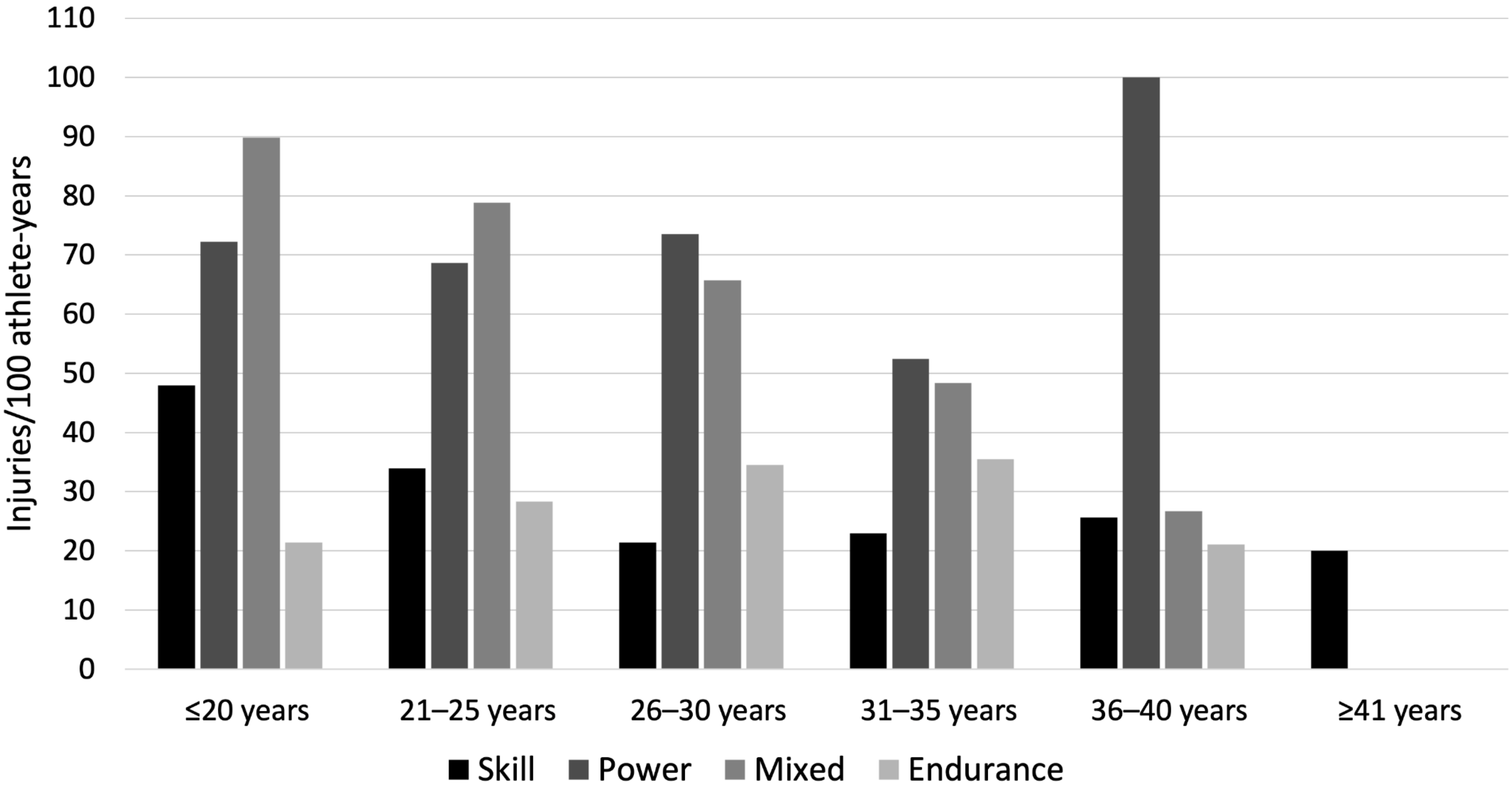

Online Resource 1. Injury incidence per age group for each sport category. For the age group ≥41 years, the incidence was zero for power, mixed, and endurance sport categories due to zero injuries and/or athlete-years. Data on age is missing for 14 athletes.
