# Supplementary material for: Swedish Olympic athletes report one injury insurance claim every second year: a 22-year insurance registry-based cohort study
Source: Knee Surg Sports Traumatol Arthrosc. 2023 Jul 15;31(10):4607–17. doi: 10.1007/s00167-023-07511-y (PMC10471666; doi:10.1007/s00167-023-07511-y)
Supplement: Supplementary file 2 — Supplementary file2 (PDF 215 KB) [file 167_2023_7511_MOESM2_ESM.pdf]

Online Resource 2. Number and percentage of injuries per subgroup for each injury type and location.

	**Sex**				**Competitive season**				**Sport category**								**Age group**[a]											
	**Male**		**Female**		**Summer sports**		**Winter sports**		**Skill**		**Power**		**Mixed**		**Endurance**		**≤20 years**		**21–25 years**		**26–30 years**		**31–35 years**		**36–40 years**		**≥41 years**	
	**n**	**%**	**n**	**%**	**n**	**%**	**n**	**%**	**n**	**%**	**n**	**%**	**n**	**%**	**n**	**%**	**n**	**%**	**n**	**%**	**n**	**%**	**n**	**%**	**n**	**%**	**n**	**%**
**Injury type**																												
Soft tissue/joint injury	203	22.6%	181	24.5%	245	22.1%	139	26.5%	41	17.2%	218	33.1%	86	18.8%	39	13.9%	63	27.5%	179	25,0%	102	20.7%	28	22,0%	10	21.7%	2	8.7%
Contusion	48	5.4%	30	4.1%	37	3.3%	41	7.8%	5	2.1%	47	7.1%	11	2.4%	15	5.3%	17	7.4%	35	4.9%	16	3.2%	6	4.7%	3	6.5%	1	4.3%
Fracture/bone injury	42	4.7%	25	3.4%	39	3.5%	28	5.3%	5	2.1%	29	4.4%	18	3.9%	15	5.3%	13	5.7%	27	3.8%	18	3.7%	5	3.9%	3	6.5%	1	4.3%
Inflammation	30	3.3%	22	3,0%	38	3.4%	14	2.7%	12	5,0%	21	3.2%	10	2.2%	9	3.2%	5	2.2%	21	2.9%	11	2.2%	9	7.1%	5	10.9%	1	4.3%
Concussion	2	0.2%	7	0.9%	3	0.3%	6	1.1%	1	0.4%	6	0.9%	1	0.2%	1	0.4%	0	0,0%	6	0.8%	3	0.6%	0	0,0%	0	0,0%	0	0,0%
Laceration/abrasion	14	1.6%	8	1.1%	12	1.1%	10	1.9%	2	0.8%	12	1.8%	4	0.9%	4	1.4%	2	0.9%	11	1.5%	6	1.2%	2	1.6%	1	2.2%	0	0,0%
Multiple injury types	5	0.6%	2	0.3%	5	0.5%	2	0.4%	1	0.4%	4	0.6%	1	0.2%	1	0.4%	0	0,0%	0	0,0%	4	0.8%	2	1.6%	1	2.2%	0	0,0%
Unspecified	553	61.6%	463	62.7%	731	65.9%	285	54.3%	171	71.8%	322	48.9%	326	71.3%	197	70.1%	129	56.3%	438	61.1%	333	67.5%	75	59.1%	23	50,0%	18	78.3%
**Injury location**																												
**Head and neck**	**22**	**2.5%**	**28**	**3.8%**	**30**	**2.7%**	**20**	**3.8%**	**6**	**2.5%**	**32**	**4.9%**	**7**	**1.5%**	**5**	**1.8%**	**2**	**0.9%**	**28**	**3.9%**	**14**	**2.8%**	**3**	**2.4%**	**3**	**6.5%**	**0**	**0.0%**
Head	12	1.3%	11	1.5%	13	1.2%	10	1.9%	1	0.4%	15	2.3%	5	1.1%	2	0.7%	1	0.4%	15	2.1%	7	1.4%	0	0.0%	0	0.0%	0	0.0%
Neck	10	1.1%	17	2.3%	17	1.5%	10	1.9%	5	2.1%	17	2.6%	2	0.4%	3	1.1%	1	0.4%	13	1.8%	7	1.4%	3	2.4%	3	6.5%	0	0.0%
**Upper limb**	**224**	**25.0%**	**160**	**21.7%**	**288**	**25.9%**	**96**	**18.3%**	**71**	**29.8%**	**159**	**24.1%**	**82**	**17.9%**	**72**	**25.6%**	**53**	**23.1%**	**173**	**24.1%**	**112**	**22.7%**	**25**	**19.7%**	**13**	**28.3%**	**8**	**34.8%**
Shoulder	106	11.8%	68	9.2%	125	11.3%	49	9.3%	43	18.1%	69	10.5%	24	5.3%	38	13.5%	24	10.5%	75	10.5%	52	10.5%	11	8.7%	6	13.0%	6	26.1%
Upper arm	2	0.2%	2	0.3%	3	0.3%	1	0.2%	0	0.0%	2	0.3%	1	0.2%	1	0.4%	1	0.4%	2	0.3%	1	0.2%	0	0.0%	0	0.0%	0	0.0%
Elbow	32	3.6%	21	2.8%	44	4.0%	9	1.7%	9	3.8%	24	3.6%	8	1.8%	12	4.3%	6	2.6%	21	2.9%	16	3.2%	5	3.9%	4	8.7%	1	4.3%
Forearm	7	0.8%	5	0.7%	6	0.5%	6	1.1%	2	0.8%	5	0.8%	2	0.4%	3	1.1%	0	0.0%	6	0.8%	4	0.8%	2	1.6%	0	0.0%	0	0.0%
Hand/wrist	75	8.4%	64	8.7%	108	9.7%	31	5.9%	16	6.7%	59	9.0%	46	10.1%	18	6.4%	22	9.6%	68	9.5%	38	7.7%	7	5.5%	3	6.5%	1	4.3%
Upper limb (unspecified)	2	0.2%	0	0.0%	2	0.2%	0	0.0%	1	0.4%	0	0.0%	1	0.2%	0	0.0%	0	0.0%	1	0.1%	1	0.2%	0	0.0%	0	0.0%	0	0.0%
**Trunk**	**153**	**17.1%**	**103**	**14.0%**	**156**	**14.1%**	**100**	**19.0%**	**52**	**21.8%**	**88**	**13.4%**	**56**	**12.3%**	**60**	**21.4%**	**30**	**13.1%**	**109**	**15.2%**	**73**	**14.8%**	**28**	**22.0%**	**11**	**23.9%**	**5**	**21.7%**
Chest/thorax	19	2.1%	16	2.2%	23	2.1%	12	2.3%	8	3.4%	9	1.4%	6	1.3%	12	4.3%	2	0.9%	14	2.0%	12	2.4%	5	3.9%	0	0.0%	2	8.7%
Spine/pelvis	131	14.6%	84	11.4%	129	11.6%	86	16.4%	41	17.2%	79	12.0%	48	10.5%	47	16.7%	27	11.8%	94	13.1%	59	12.0%	22	17.3%	10	21.7%	3	13.0%
Abdomen	3	0.3%	3	0.4%	4	0.4%	2	0.4%	3	1.3%	0	0.0%	2	0.4%	1	0.4%	1	0.4%	1	0.1%	2	0.4%	1	0.8%	1	2.2%	0	0.0%
**Lower limb**	**482**	**53.7%**	**439**	**59.5%**	**621**	**55.9%**	**300**	**57.1%**	**104**	**43.7%**	**373**	**56.6%**	**304**	**66.5%**	**140**	**49.8%**	**141**	**61.6%**	**401**	**55.9%**	**284**	**57.6%**	**68**	**53.5%**	**18**	**39.1%**	**9**	**39.1%**
Thigh/hip/groin	82	9.1%	73	9.9%	121	10.9%	34	6.5%	12	5.0%	46	7.0%	74	16.2%	23	8.2%	16	7.0%	63	8.8%	60	12.2%	9	7.1%	4	8.7%	3	13.0%
Knee	220	24.5%	176	23.8%	233	21.0%	163	31.0%	49	20.6%	216	32.8%	76	16.6%	55	19.6%	66	28.8%	187	26.1%	103	20.9%	30	23.6%	5	10.9%	5	21.7%
Lower leg	54	6.0%	58	7.9%	85	7.7%	27	5.1%	11	4.6%	20	3.0%	57	12.5%	24	8.5%	12	5.2%	37	5.2%	44	8.9%	14	11.0%	4	8.7%	1	4.3%
Foot/ankle	120	13.4%	129	17.5%	178	16.0%	71	13.5%	30	12.6%	89	13.5%	93	20.4%	37	13.2%	46	20.1%	108	15.1%	76	15.4%	14	11.0%	5	10.9%	0	0.0%
Lower limb (unspecified)	6	0.7%	3	0.4%	4	0.4%	5	1.0%	2	0.8%	2	0.3%	4	0.9%	1	0.4%	1	0.4%	6	0.8%	1	0.2%	1	0.8%	0	0.0%	0	0.0%
**Multiple locations**	**10**	**1.1%**	**7**	**0.9%**	**10**	**0.9%**	**7**	**1.3%**	**5**	**2.1%**	**4**	**0.6%**	**4**	**0.9%**	**4**	**1.4%**	**1**	**0.4%**	**5**	**0.7%**	**8**	**1.6%**	**1**	**0.8%**	**1**	**2.2%**	**1**	**4.3%**
**Unspecified**	**6**	**0.7%**	**1**	**0.1%**	**5**	**0.5%**	**2**	**0.4%**	**0**	**0.0%**	**3**	**0.5%**	**4**	**0.9%**	**0**	**0.0%**	**2**	**0.9%**	**1**	**0.1%**	**2**	**0.4%**	**2**	**1.6%**	**0**	**0.0%**	**0**	**0.0%**

[a]Data on age is missing for 14 athletes
