# Supplementary material for: Swedish Olympic athletes report one injury insurance claim every second year: a 22-year insurance registry-based cohort study
Source: Knee Surg Sports Traumatol Arthrosc. 2023 Jul 15;31(10):4607–17. doi: 10.1007/s00167-023-07511-y (PMC10471666; doi:10.1007/s00167-023-07511-y)
Supplement: Supplementary file 3 — Supplementary file3 (PDF 155 KB) [file 167_2023_7511_MOESM3_ESM.pdf]

Online Resource 3. Number and percentage of injuries per injury type for each location.

	Soft tissue/joint injury		Contusion		Fracture/bone injury		Inflammation		Concussion		Laceration/abrasion		Multiple injury types		Unspecified	
	**n**	**%**	**n**	**%**	**n**	**%**	**n**	**%**	**n**	**%**	**n**	**%**	**n**	**%**	**n**	**%**
**Head and neck**	**15**	**3.9%**	**2**	**2.6%**	**2**	**3.0%**	**0**	**0.0%**	**9**	**100%**	**6**	**27.3%**	**1**	**14.3%**	**15**	**1.5%**
Head	0	0.0%	2	2.6%	2	3.0%	0	0.0%	9	100%	6	27.3%	1	14.3%	3	0.3%
Neck	15	3.9%	0	0.0%	0	0.0%	0	0.0%	N/A	N/A	0	0.0%	0	0.0%	12	1.2%
**Upper limb**	**36**	**9.4%**	**34**	**43.6%**	**29**	**43.3%**	**20**	**38.5%**	**N/A**	**N/A**	**2**	**9.1%**	**0**	**0.0%**	**263**	**25.9%**
Shoulder	18	4.7%	18	23.1%	6	9.0%	5	9.6%	N/A	N/A	2	9.1%	0	0.0%	125	12.3%
Upper arm	0	0.0%	0	0.0%	0	0.0%	1	1.9%	N/A	N/A	0	0.0%	0	0.0%	3	0.3%
Elbow	1	0.3%	3	3.8%	3	4.5%	9	17.3%	N/A	N/A	0	0.0%	0	0.0%	37	3.6%
Forearm	0	0.0%	0	0.0%	4	6.0%	1	1.9%	N/A	N/A	0	0.0%	0	0.0%	7	0.7%
Hand/wrist	17	4.4%	13	16.7%	16	23.9%	4	7.7%	N/A	N/A	0	0.0%	0	0.0%	89	8.8%
Upper limb (unspecified)	0	0.0%	0	0.0%	0	0.0%	0	0.0%	N/A	N/A	0	0.0%	0	0.0%	2	0.2%
**Trunk**	**128**	**33.3%**	**12**	**15.4%**	**7**	**10.4%**	**3**	**5.8%**	**N/A**	**N/A**	**0**	**0.0%**	**0**	**0.0%**	**106**	**10.4%**
Chest/thorax	2	0.5%	0	0.0%	5	7.5%	1	1.9%	N/A	N/A	0	0.0%	0	0.0%	27	2.7%
Spine/pelvis	125	32.6%	12	15.4%	2	3.0%	2	3.8%	N/A	N/A	0	0.0%	0	0.0%	74	7.3%
Abdomen	1	0.3%	0	0.0%	0	0.0%	0	0.0%	N/A	N/A	0	0.0%	0	0.0%	5	0.5%
**Lower limb**	**205**	**53.4%**	**26**	**33.3%**	**28**	**41.8%**	**28**	**53.8%**	**N/A**	**N/A**	**10**	**45.5%**	**0**	**0.0%**	**624**	**61.4%**
Thigh/hip/groin	28	7.3%	8	10.3%	2	3.0%	2	3.8%	N/A	N/A	0	0.0%	0	0.0%	115	11.3%
Knee	110	28.6%	12	15.4%	3	4.5%	12	23.1%	N/A	N/A	6	27.3%	0	0.0%	253	24.9%
Lower leg	15	3.9%	2	2.6%	8	11.9%	9	17.3%	N/A	N/A	1	4.5%	0	0.0%	77	7.6%
Foot/ankle	52	13.5%	4	5.1%	15	22.4%	5	9.6%	N/A	N/A	3	13.6%	0	0.0%	170	16.7%
Lower limb (unspecified)	0	0.0%	0	0.0%	0	0.0%	0	0.0%	N/A	N/A	0	0.0%	0	0.0%	9	0.9%
**Multiple locations**	**0**	**0.0%**	**4**	**5.1%**	**0**	**0.0%**	**1**	**1.9%**	**N/A**	**N/A**	**1**	**4.5%**	**3**	**42.9%**	**8**	**0.8%**
**Unspecified**	**0**	**0.0%**	**0**	**0.0%**	**1**	**1.5%**	**0**	**0.0%**	**N/A**	**N/A**	**3**	**13.6%**	**3**	**42.9%**	**0**	**0.0%**
