# Supplementary material for: Swedish Olympic athletes report one injury insurance claim every second year: a 22-year insurance registry-based cohort study
Source: Knee Surg Sports Traumatol Arthrosc. 2023 Jul 15;31(10):4607–17. doi: 10.1007/s00167-023-07511-y (PMC10471666; doi:10.1007/s00167-023-07511-y)
Supplement: Supplementary file 4 — Supplementary file4 (PDF 109 KB) [file 167_2023_7511_MOESM4_ESM.pdf]

Online Resource 4. Number of athlete-years per sport category for each age group.

	≤20 years[a]	21–25 years[a]	26–30 years[a]	31–35 years[a]	36–40 years[a]	≥41 years[a]
**Sport category**	**Athlete-years[b]**	**Athlete-years[b]**	**Athlete-years[b]**	**Athlete-years[b]**	**Athlete-years[b]**	**Athlete-years[b]**
Skill sports	50.0	197.5	280.0	152.5	113.0	115.0
Power sports	155.0	460.5	262.5	51.5	11.0	1.5
Mixed sports	83.5	279.0	210.0	45.5	7.5	0
Endurance sports	84.0	403.0	295.5	121.0	19.0	0

[a]Data on age is missing for 14 athletes

[b]Athlete-years are defined as the number of years an athlete participated in Top and Talent. If an athlete only participated half a year (spring or autumn) they were given a value of 0.5
