# Supplementary material for: Swedish Olympic athletes report one injury insurance claim every second year: a 22-year insurance registry-based cohort study
Source: Knee Surg Sports Traumatol Arthrosc. 2023 Jul 15;31(10):4607–17. doi: 10.1007/s00167-023-07511-y (PMC10471666; doi:10.1007/s00167-023-07511-y)
Supplement: Supplementary file 6 — Supplementary file6 (PDF 565 KB) [file 167_2023_7511_MOESM6_ESM.pdf]

Appendix 3: STROBE-SIIS (Sports Injury and Illness Surveillance) Statement 1.0—Checklist of items for the reporting of observational studies on injury and illness in sports

**Item**	**Item No**	**Recommendation from the STROBE Statement**	**STROBE-SIIS Extension** +	**Source of rationale for item from the consensus statement and where to find further details**	
**Title and abstract**	1	(*a*) Indicate the study's design with a commonly used term in the title or the abstract			
		(*b*) Provide in the abstract an informative and balanced summary of what was done and what was found	SIIS-1.1. Include information on the sport, athlete population (sex, age, geographic region) and level of competition.<br>SIIS-1.2. Include the duration of observation (e.g. one season, one year, multiple years).	SIIS-1.1. 'Study population characteristics'<br>SIIS-1.2. 'Capturing and reporting athlete exposure'	**Page 1**<br>**Page 1**
**Introduction**					
Background/rationale	2	Explain the scientific background and rationale for the investigation being reported			
Objectives	3	State specific objectives, including any pre-specified hypotheses	SIIS-3.1. State whether study was registered. Identify the registration number and database used.<br>SIIS-3.2. State the specific purpose of your study (e.g. to describe the injury burden associated with Olympic level rowing)	SIIS-3.1. 'Reporting guidelines—STROBE Sports Injury and Illness Surveillance (STROBE-SIIS)'<br>SIIS-3.2 Throughout consensus statement	**NA**<br>**Page 3**
**Methods**					
Study design	4	Present key elements of study design early in the paper	SIIS-4.1. Clearly specify which health problems are being observed.	SIIS-4.1. 'Defining and classifying health problems'	**Page 5**

Item	Item No	Recommendation from the STROBE Statement	STROBE-SIIS Extension +	Source of rationale for item from the consensus statement and where to find further details	
			SIIS-4.2. State explicitly which approach was used to record the health problem data, including all outcome measures or tools	SIIS-4.2. 'Data collection methods'	**Page 4-5**
			SIIS-4.3. State explicitly which coding system was used to classify the health problems (e.g. OSIICS, SMDCS, ICD, etc.)	SIIS-4.3. 'Classifying sports injury and illness diagnoses'	**Page 4**
			SIIS-4.4. Where relevant, clearly describe how athletes were categorised. Variables to consider could include the type of athlete and/or sport, the environment in which the sport occurs (e.g. type of course or playing area), the typical duration of the sport, the degree of physical contact permitted in the sport, and the equipment permitted.	SIIS-4.4. 'Study population characteristics'	**Page 5-6**
Setting	5	Describe the setting, locations, and relevant dates, including periods of recruitment, exposure, follow-up, and data collection	SIIS-5.1. Describe the location, level of play, dates of observation and data collection methods (i.e. who, what, where).	SIIS-5.1. 'Study population characteristics'	**Page 4**
			SIIS-5.2. Specify the dates of the surveillance period and how the data were handled when the study covered more than one season/calendar year.	SIIS-5.2. 'Capturing and reporting athlete exposure'	**Page 4-5**
			SIIS-5.3. Define whether the health problem data were collected prospectively or retrospectively.	SIIS-5.3. 'Capturing and reporting athlete exposure' and 'Data collection methods'	**Page 4**

Item	Item No	Recommendation from the STROBE Statement	STROBE-SIIS Extension +	Source of rationale for item from the consensus statement and where to find further details	
Participants	6	(*a*) *Cohort study*—Give the eligibility criteria, and the sources and methods of selection of participants. Describe methods of follow-up<br>*Case-control study*—Give the eligibility criteria, and the sources and methods of case ascertainment and control selection. Give the rationale for the choice of cases and controls<br>*Cross-sectional study*—Give the eligibility criteria, and the sources and methods of selection of participants	SIIS-6.1. Define the population of athletes and how they were selected and recruited.	SIIS-6.1. 'Data collection methods' and 'Study population characteristics'	**Page 4**
		(*b*) *Cohort study*—For matched studies, give matching criteria and number of exposed and unexposed<br>*Case-control study*—For matched studies, give matching criteria and the number of controls per case			**NA**
Variables	7	Clearly define all outcomes, exposures, predictors, potential confounders, and effect modifiers. Give diagnostic criteria, if applicable	SIIS-7.1. Justify why you measured your primary and secondary outcomes of interest in the specific way chosen.<br>SIIS-7.2. Describe the method for identifying your health problem outcome of interest.	SIIS-7.1. 'Defining and classifying health problems'<br>SIIS-7.2. 'Defining and classifying health problems'	**Page 4-5**<br>**Page 4-5**
Data sources/ measurement	8*	For each variable of interest, give sources of data and details of methods of assessment (measurement). Describe	SIIS-8.1. Specify who collected/reported the data for the	SIIS-8.1. 'Classifying sports injury and illness diagnoses' and 'Data collection methods'	**Page 4**

Item	Item No	Recommendation from the STROBE Statement	STROBE-SIIS Extension +	Source of rationale for item from the consensus statement and where to find further details	
		comparability of assessment methods if there is more than one group	study and their qualifications (e.g. qualified doctor, data analyst, etc.).	SIIS-8.2. 'Classifying sports injury and illness diagnoses'	
			SIIS-8.2. Specify who coded the data for the study and their qualifications (e.g. qualified doctor, data analyst, etc.). In many instances, this will not be the same as SIIS-8.1.	SIIS-8.3. 'Data collection methods'<br>SIIS-8.4. 'Relationship to sports activity' and 'Capturing and reporting athlete exposure'<br>SIIS-8.5. 'Relationship to sports activity' and 'Capturing and reporting athlete exposure'	**Page 4**
			SIIS-8.3. Specify the direct methods used to collect the data, and the use of physical documents or an electronic tools. If extracting information from existing sources, specify the data source.		**Page 4-5**
			SIIS-8.4. Specify the timing of and window for data collection (e.g. day health problem occurred or following day). Specify the frequency of data collection (e.g. daily, weekly, monthly).		**Page 4**
			SIIS-8.5. Report the duration of surveillance (e.g. tournament, season, whole year, playing career).		**Page 4**
Bias	9	Describe any efforts to address potential sources of bias	SIIS-9.1. Clearly report any validation or reliability assessment of the data collection of tools.	SIIS-9.1. 'Data collection methods'<br>SIIS-9.2. 'Data collection methods'	**NA**
			SIIS-9.2. Formally acknowledge any potential biases in associated with the data collection method (e.g. self-		**Page 10**

Item	Item No	Recommendation from the STROBE Statement	STROBE-SIIS Extension +	Source of rationale for item from the consensus statement and where to find further details	
			report, recall bias, reporting by non-medically trained staff, etc.)		
Study size	10	Explain how the study size was arrived at			
Quantitative variables	11	Explain how quantitative variables were handled in the analyses. If applicable, describe which groupings were chosen and why	SIIS-11.1 Explain in detail how multiple injuries/illness episodes are handled both in individual athletes and across athletes/surveillance periods.<br>SIIS-11.2. Specify how injury severity was calculated.	SIIS-11.1. 'Multiple events and health problems' and 'Subsequent, recurrent and/or exacerbation of health problems'<br>SIIS-11.2. 'Severity of health problems'	**Page 5**<br>**NA**
Statistical methods	12	(*a*) Describe all statistical methods, including those used to control for confounding	SIIS-12.1. Specify how exposure to risk has been adjusted for and specify the units (e.g. per participant, per athlete exposure, etc.).<br>SIIS-12.2 Specify how relevant risk measures (incidence, prevalence, etc.) were calculated.<br>SIIS-12.3. When relevant to the study aim, specify how injury burden was calculated and analysed.	SIIS-12.1. 'Capturing and reporting athlete exposure'<br>SIIS-12.2 'Expressing risk'<br>SIIS-12.3. 'Burden of health problems'	**Page 5**<br>**Page 5**<br>**NA**
		(*b*) Describe any methods used to examine subgroups and interactions			
		(*c*) Explain how missing data were addressed	SIIS-12.4. For studies reporting multiple health problems, state clearly how these were handled (e.g. time to the first injury only, ignoring	SIIS-12.4. 'Multiple health problems' and 'Subsequent, recurrent and/or exacerbation of injury/illness'<br>SIIS-12.5. 'Capturing and reporting athlete exposure'	**Page 5-6**

Item	Item No	Recommendation from the STROBE Statement	STROBE-SIIS Extension [+]	Source of rationale for item from the consensus statement and where to find further details	
			subsequent return to play and re-injuries, or modelling of all injuries).		
			SIIS-12.5. Explain how/if athletes not included at outset (e.g. those already injured) were handled in the analyses.		**NA**
		(*d*) *Cohort study*—If applicable, explain how loss to follow-up was addressed<br>*Case-control study*—If applicable, explain how matching of cases and controls was addressed<br>*Cross-sectional study*—If applicable, describe analytical methods taking account of sampling strategy	SIIS-12.6. In longitudinal studies, it is particularly important to explain how athlete follow-up has been managed. For example, what happened if a player was transferred to another team or has been censored (for those no longer part of the study due to removal during the observation period). Censoring can occur when athletes are removed due to transfer out of the team/study, injury/illness or due to study design.	SIIS-12.6. 'Capturing and reporting athlete exposure'	**Page 5**
		(*e*) Describe any sensitivity analyses			
**Results**					
Participants	13*	(a) Report numbers of individuals at each stage of study—e.g. numbers potentially eligible, examined for eligibility, confirmed eligible, included in the study, completing follow-up, and analysed	SIIS-13.1. Clearly state the number of athletes followed-up, the number (and %) of those with the health problem and the number of problems reported among them (a median number of problems per affected athlete could be useful).	SIIS-13.1. 'Multiple health problems'<br>SIIS-13.2. 'Multiple health problems' and Expressing risk'	**Page 6**
			SIIS-13.2. For studies over multiple seasons/years, report the total		**Fig. 1**

Item	Item No	Recommendation from the STROBE Statement	STROBE-SIIS Extension +	Source of rationale for item from the consensus statement and where to find further details	
			numbers of health problems for each year and numbers common to each period.		
		(b) Give reasons for non-participation at each stage	SIIS-13.3. Report how athletes removed (e.g. due to transfer of teams or time-out due to injury or illness) impact upon data at key data collection/reporting points, ideally with a flow diagram	SIIS-13.3. Throughout consensus statement	**NA**
Descriptive data	14*	(a) Give characteristics of study participants (e.g. demographic, clinical, social) and information on exposures and potential confounders	SIIS-14.1. Include detail on the level of competition being observed (e.g. by age levels, skill level, sex, etc.).	SIIS-14.1. 'Study population characteristics'	**Page 4**
		(b) Indicate number of participants with missing data for each variable of interest			
		(c) *Cohort study*—Summarise follow-up time (e.g. average and total amount)			
Outcome data	15*	*Cohort study*—Report numbers of outcome events or summary measures over time	SIIS-15.1. In many observational studies, individuals will sustain more than one health problem over the surveillance period. Take care to ensure descriptive data representing both the number of health problems and the number of athletes affected. It is important to represent effectively both the analysis and reporting of correct units for frequency data, i.e. the % of affected	SIIS-15.1. 'Multiple health problems' and 'Subsequent, recurrent and/or exacerbation of injury/illness'	**Page 6 Table 3-5**

Item	Item No	Recommendation from the STROBE Statement	STROBE-SIIS Extension [+]	Source of rationale for item from the consensus statement and where to find further details	
			athletes or the % of injuries, body regions, etc.		
		Case-control study—Report numbers in each exposure category, or summary measures of exposure			
		Cross-sectional study—Report numbers of outcome events or summary measures			
Main results	16	(*a*) Give unadjusted estimates and, if applicable, confounder-adjusted estimates and their precision (e.g. 95% confidence interval). Make clear which confounders were adjusted for and why they were included	SIIS-16.1. Report exposure-adjusted incidence or prevalence measures with appropriate confidence intervals when presenting risk measures.<br>SIIS-16.2. Report details of interest, such as mode of onset	SIIS-16.1. 'Expressing risk'<br>SIIS-16.2. 'Relationship to sports activity', 'Mode of onset—injury', Mode of onset—illness' and 'Classifying the mechanism of injury'	**Page 6-7**<br>**Table 3-5**<br>**Page 7**
		(*b*) Report category boundaries when continuous variables were categorized			
		(*c*) If relevant, consider translating estimates of relative risk into absolute risk for a meaningful time period			
Other analyses	17	Report other analyses done—e.g. analyses of subgroups and interactions, and sensitivity analyses	SIIS-17.1 Report injury diagnosis information, including region and tissue type in tabular form.	SIIS-17.1. 'Defining and classifying health problems'	**Table 4-5**<br>**Online resource 2-3**
**Discussion**					
Key results	18	Summarise key results with reference to study objectives			
Limitations	19	Discuss limitations of the study, taking into account sources of potential bias or	SIIS-19.1. Discuss limitations in the data collection and coding procedures adopted, including in	SIIS 19.1. 'Data collection methods' and 'Expressing risk'	**Page 10**

Item	Item No	Recommendation from the STROBE Statement	STROBE-SIIS Extension +	Source of rationale for item from the consensus statement and where to find further details	
		imprecision. Discuss both direction and magnitude of any potential bias	relation to any risk measures calculated.		
Interpretation	20	Give a cautious overall interpretation of results considering objectives, limitations, multiplicity of analyses, results from similar studies, and other relevant evidence			
Generalisability	21	Discuss the generalisability (external validity) of the study results	SIIS-21.1. Discuss the generalizability of the athlete study population, and health problem sub-groups of interest, to broader athlete groups.	SIIS-21.2. 'Relationship to sports activity' and 'Study population characteristics'	**Page 10**
**Other information**					
Funding	22	Give the source of funding and the role of the funders for the present study and, if applicable, for the original study on which the present article is based			
Ethics	23		SIIS-23.1. Outline how individual athlete data privacy and confidentiality considerations were addressed, in line with the Declaration of Helsinki.	SIIS-23.1. 'Research ethics and data security'	**Page 4**

Note: The STROBE-SIIS checklist with additional sports epidemiology annotations should be used in conjunction with the original STROBE guideline (freely available on the Web sites of PLoS Medicine at http://www.plosmedicine.org/, Annals of Internal Medicine at http://www.annals.org/, and Epidemiology at http://www.epidem.com/). Information on the STROBE Initiative is available at www.strobe-statement.org.

+For brevity, the phrase health problem is used here to encompass both injury and illness.

*Give information separately for cases and controls in case-control studies and, if applicable, for exposed and unexposed groups in cohort and cross-sectional studies.

^^ Where there is a blank cell in this column, there are no specific additional reporting requirements for sports injury and illness surveillance over what is already covered in the original STROBE checklist.

Reference

1. Orchard O, Meeuwisse W, Derman W, et al. Refinement and presentation of the Calgary Sport Medicine Diagnostic Coding System (SMDSC) and the Orchard Sport Injury & Illness Classification System (OSIICS). *Br J Sports Med* In preparation
